# Imaging cochlear implantation with round window insertion in human temporal bones and cochlear morphological variation using high-resolution cone beam CT

**DOI:** 10.3109/00016489.2014.993090

**Published:** 2015-02-13

**Authors:** Jing Zou, Jaakko Lähelmä, Juha Koivisto, Anandhan Dhanasingh, Claude Jolly, Antti Aarnisalo, Jan Wolff, Ilmari Pyykkö

**Affiliations:** ^a^ ^1^Hearing and Balance Research Unit, Field of Oto-laryngology, School of Medicine, University of Tampere, Tampere, Finland; ^b^ ^2^PlanmecaOy, Helsinki, Finland; ^c^ ^3^Department of Physics, University of Helsinki, Helsinki, Finland; ^d^ ^4^MED-EL Medical Electronics, Innsbruck, Austria; ^e^ ^5^Department of Otorhinolaryngology-Head and Neck Surgery, Helsinki University Central Hospital, Helsinki, Finland

**Keywords:** Cochlear implant, imaging, computed tomography, human anatomy, surgery

## Abstract

*Conclusions:* The present experimental set-up of high spatial resolution cone-beam computed tomography (CBCT) showed advantages of demonstrating the critical landmarks of the cochlea in identifying the position of intracochlear electrode contacts and has the potential for clinical application in cochlear implant (CI) surgery. *Objective:* To evaluate a newly developed CBCT system in defining CI electrode array in human temporal bone and cochlear morphological variation. *Methods:* Standard electrode, flexible tip electrode (Flex28), and an experimental electrode array with 36 contacts from MED-EL were implanted into the cochleae of six human temporal bones through an atraumatic round window membrane insertion. The cochleae were imaged with 900 frames using an experimental set-up based on a CBCT scanner installed with Superior SXR 130-15-0.5 X-ray tube in combination with filtration of copper and aluminum. *Results:* In all temporal bones, the landmarks of the cochlea, modiolus, osseous spiral lamina, round window niche, and stapes were demonstrated at an average level of 3.4–4.5. The contacts of electrode arrays were clearly shown to locate in the scala tympani. There was a linear correlation between the ‘A’ value and cochlea height, and between the A value and actual electrode insertion length for the first 360° insertion depth.

## Introduction

Cone-beam computed tomography (CBCT) is going to play a key role in cochlear implantation, which provides functional restoration of hearing in individuals with profound hearing impairment, in both planning the implantation (selection of electrode model and designing the surgical procedure) before surgery and quality control (confirmation of electrode array position accurately in the cochlear scala) during the surgery. CBCT has the advantage over multi-detector CT (MDCT) of fast data acquisition, which is less than a minute versus several minutes for MDCT, low-dose exposure of the subject, small metallic artefact, and a relatively low equipment purchase price. Husstedt et al. first demonstrated electrode–modiolus relationship after cochlear implantation in isolated temporal bones in 2002 using a C-arm-based radiographic device [1]. After that, Gupta et al. in 2004 reported an experimental flat-panel high-spatial resolution volume CT for temporal bone imaging using a smaller detector element and acquired a total of 900 cone-beam projections under a field of view of 15.5 cm [2]. Recently, Pearl et al. reported high-resolution secondary reconstructions with the use of flat-panel CT to assess the cochlear implant (CI) location in the patients postoperatively [3]. However, the fine structures inside the cochlea, which are critical landmarks to identify the position of cochlear electrode arrays intracochlearly, were not demonstrated in any of those studies. In addition to the disadvantage of high-dosage exposure of the subject, MDCT did not display satisfying images of the CI in the cochlea with respect to spatial resolution and metal artefacts [4, 5].

Planmeca Oy (Helsinki, Finland) has developed software that is capable of creating 3D reconstruction of 900 frames of two-dimensional (2D) images using a Superior SXR 130-15-0.5 X-ray tube (Superior X-ray Tube Co., Woodstock, IL, USA). A combination of copper and aluminum filters was used to adjust the contrast of images. The acquired images were used in evaluating certain important cochlear measurements, which has the potential for application in cochlear implantation. For instance, basal turn diameter is selected by several research groups as a reference in correlating with the cochlear length, which is useful in choosing the right electrode array [6, 7]. The distance between the electrode array contacts and the modiolus wall is an indicator in the case of free-fitting lateral wall electrodes that will predict the fields of excitation to the nearby neural tissue, as the free-fitting lateral wall electrodes are located far from the modiolus wall in contrast to perimodiolar electrodes [8]. The electrode insertion depth expressed as insertion angle is relevant in estimating the percentage of cochlear coverage with the electrical stimulation that was reportedly functioning as an indicator of the performance of the profoundly deaf patient postoperatively, showing the correlation of electrode deep insertion with speech perception [9]. The high quality images acquired with the new set-up are helpful in evaluating the potential migration of the electrode array from the scala tympani to the scala media, which is a sign of intracochlear trauma during electrode insertion into the cochlea.

This article mainly reports on the efficacy of a new CBCT imaging set-up in visualizing the intracochlear fine structures in cochlear implantation with insignificant interference of metal artefacts and the application in these aforementioned critical cochlear assessments.

## Material and methods

### Electrodes and temporal bone procedure

Six human temporal bones were used in imaging. They all were donated for scientific use at the University of Tampere and fulfilled the requirements of the Helsinki Declaration for ethical use of human material.

**Figure 1.**
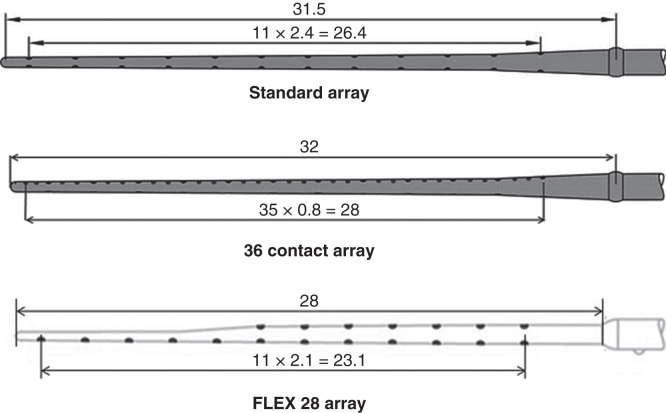
**Electrode array types.**

The following electrodes were provided by MED-EL, Innsbruck, Austria (Figure 1, Table I). The standard electrode has 12 pairs of stimulating contacts made of platinum with an active stimulation length of 26.4 mm (2.4 mm contact separation). Flex28 is thinner and more flexible than the standard electrode and has seven pairs of stimulating contacts in the basal part and a single line of five contacts in the apical part with an active stimulation length of 23.1 mm. The third type is an experimental electrode array with 36 stimulating contacts arranged in a single line and extended with an active stimulation length of 28 mm. More details on the electrode dimensions are given in Table I. Six formaldehyde-preserved human temporal bones were implanted with these electrodes in the Temporal Bone Lab of Tampere University Hospital through an atraumatic round window membrane (RWM) insertion. The round window niche was exposed through a posterior tympanotomy and mainly the anterior bony overhang of the round window niche was removed. A transverse incision was made across the RWM and a defined electrode was carefully inserted into the scala tympani through the incised RWM. The rest of the electrode was coiled inside the drilled mastoid cavity and packed with tissue pieces.

**Table I.** **Technical specifications of the electrode types.**

Factor	Standard array	Experimental array with 36 contacts	Flex28
Active stimulation length	26.4 mm	28	23.1
Number of contacts	24 (12 pairs)	36 (one line of contacts)	19 (7 pairs and 5 in a single line)
Contact separation	2.4 mm	0.8 mm	2.1 mm
Diameter at basal end	1.3 mm	1.3	0.8
Dimensions at apical end	0.5 mm	0.5	Oval-shaped (0.48/0.36)
Number of operations	3	1	2

### CBCT imaging of temporal bones

Images were obtained with an experimental set-up based on a CBCT scanner (Planmeca Oy) using a Superior SXR 130-15-0.5 X-ray tube (Superior X-ray Tube Co.). The novelty of the experimental set-up is that, to reduce potential motion artifact generated by the rotating C-arm during scanning, the multiple frames of images were obtained by rotating the sample stage using a step motor instead of rotating the C-arm as in the standard CBCT equipment. A rotating sample stage (round plate in the center) is much more stable than a rotating C-arm. Imaging parameters used in this study were: number of frames 900, tube voltage 88 kV, tube current 11 mA, exposure length per frame 50 ms, filtration 0.5 mm copper + 2.5 mm aluminum, source-to-image distance 1 m, magnification factor 1.17, voxel size 0.1 mm, and field of view (FOV) 60 × 60 mm.

### Evaluation of image quality

By making a non-linear adjustment to the pixel values using a predetermined S-shaped curve (γ curve) of the original images that were displayed using the Romexis Viewer Demo program (Planmeca Oy), the critical structures of the inner ear and electrode were demonstrated with insignificant interference from metal artefacts. For image quality evaluation, each image was independently analyzed by three senior otologists (J.Z., I.P., and A.A.) and two senior engineers (J.K. and J.L.). Images were processed using a Romexis Viewer Demo program (Planmeca Oy) to demonstrate the critical structures of the middle and inner ears and electrode. Readers were blinded to the imaging parameters during the evaluation of each scan. To provide a quantitative assessment of image quality, the cochlea (the normal contour and different turns), modiolus (presence and borders), osseous spiral lamina (presence and borders), round window niche (presence and borders), and stapes (presence of all parts including the head, footplate, and anterior and posterior crura) were evaluated using a rating system from 5 to 1 in descending order: 5, very good delineation of structures and excellent quality; 4, clear delineation of structures and good image quality; 3, anatomic structures still fully assessable in all parts and acceptable image quality; 2, structures identified but no details assessable and results in insufficient image quality; 1, anatomic structures not identifiable due to poor image quality. The osseous spiral lamina is the landmark to identify the location of the electrode array inside the cochlea and has been utilized for image quality evaluation in MDCT scanning, which was rated as: 0 = not visible; 1 = visible in most parts of the cochlea; 2 = good delineation of the lamina [10]. In the present rating system, level 5 was included to indicate very good delineation of structures because the present experimental set-up of CBCT provided a chance to demonstrate the cochlear structures with super quality. Level 3 was introduced to show more details between levels 0 and 1 of the reported rating system [10].

### Cochlear measurements

**Figure 2.**
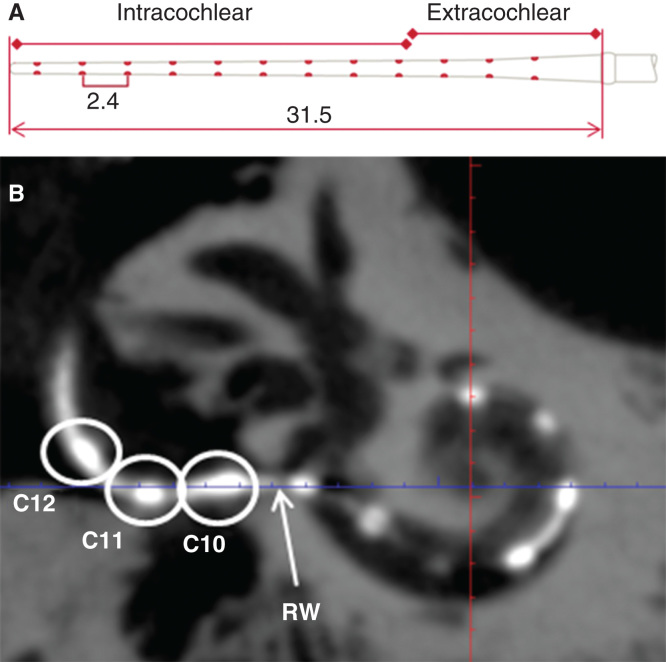
**Measurement of full insertion depth of the electrode. A standard electrode array with the measures (mm) is shown in (A). On the cone-beam computed tomography (CBCT) image (B), three contacts (C10–C12) were outside the round window (RW) and the intracochlear measures started from the RW.**

The basal turn diameter abbreviated as ‘A’ was measured as reported in the literature, the longest distance between the center of the RWM and opposite lateral wall of the basal turn along the center of the modiolus [6]. The height of the cochlea was measured from the lower base of the basal turn to the upper base of the apical turn. The actual insertion depth of the electrode was measured by following the central axis of the electrode from the round window opening to the first 360° of insertion angle (complete basal turn) and continued to the apical tip of the electrode when extended beyond the basal turn (named as full insertion depth of the electrode) (Figure 2), and the corresponding insertion angle for the full insertion depth of the electrode was determined as reported by Trieger et al. [11]. Distances between the center of the electrode contacts and modiolus wall were also measured. The correlations between ‘A’ value and the other cochlear dimensions were analyzed. 3D reconstruction with bony threshold was carried out to demonstrate the spatial relation between the electrode contacts and modiolus.

## Results

### Image quality

**Figure 3.**
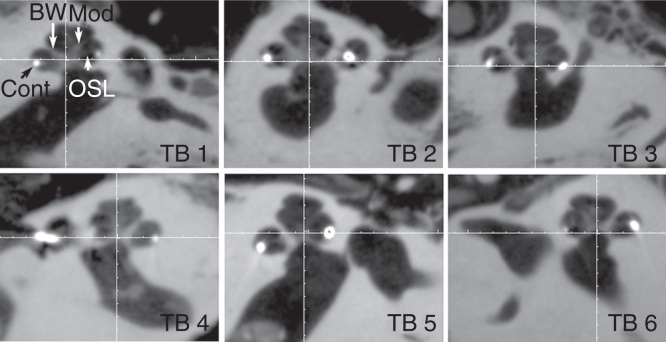
**Fine cochlear structures demonstrated by cone-beam computed tomography (CBCT). All specified landmarks were clearly identified in all six temporal bones (TB). BW, bony wall; Cont, contours of cochlear implant; Mod, modiolus; OSL, osseous spiral lamina.**

In all temporal bones imaged with the present experimental set-up, the landmarks of the cochlea, modiolus, osseous spiral lamina, round window niche, and stapes were demonstrated at an average level of 3.4–4.5 (Table II). The osseous spiral lamina was fully assessable in all parts with relatively good image quality (average level of 3.4) in the present study, but was not identified in images scanned by MDCT [10]. The contacts of electrode arrays were clearly shown to locate in the scala tympani (below the osseous spiral lamina) (Figure 3).

**Table II.** **Image quality demonstrated by average levels of five evaluators using a rating system from 5 to 1 in descending order.**

Landmarks	Levels
Cochlea	3.8
Osseous spiral lamina	3.4
Modiolus	3.9
Stapes	4.5
Round window niche	4.3
Oval window	4.4

Level: 5, very good delineation of structures and excellent quality; 4, clear delineation of structures and good image quality; 3, anatomic structures still fully assessable in all parts and acceptable image quality; 2, structures identified but no details assessable and results in insufficient image quality; 1, anatomic structures not identifiable due to poor image quality.

**Figure 4.**
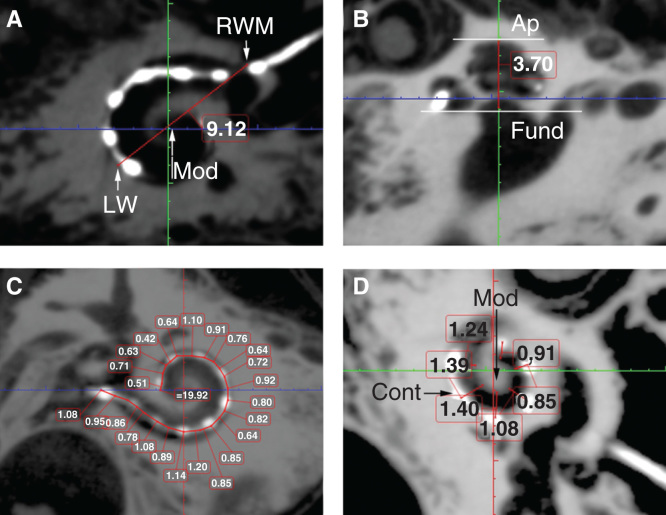
**Critical cochlear assessments on cone-beam computed tomography (CBCT) images. ‘A’ value (A), cochlear height (B), insertion depth for the first 360° insertion angle (C), and distance between the electrode contacts and the modiolus (D) are demonstrated (in mm). Ap, apex; Cont, contours of cochlear implant; Fund, fundus; LW, lateral wall; Mod, modiolus; RWM, round window membrane.**

**Figure 5.**
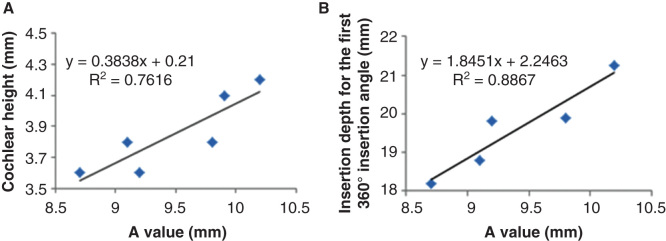
**Correlations between ‘A’ value and cochlear implant electrode array insertion parameters measured on cone-beam computed tomography (CBCT) images. Good correlations appeared between A value and cochlear height shown (A), and between A value and insertion depth for the first 360° insertion angle (B). Sample 2 was removed.**

**Figure 6.**
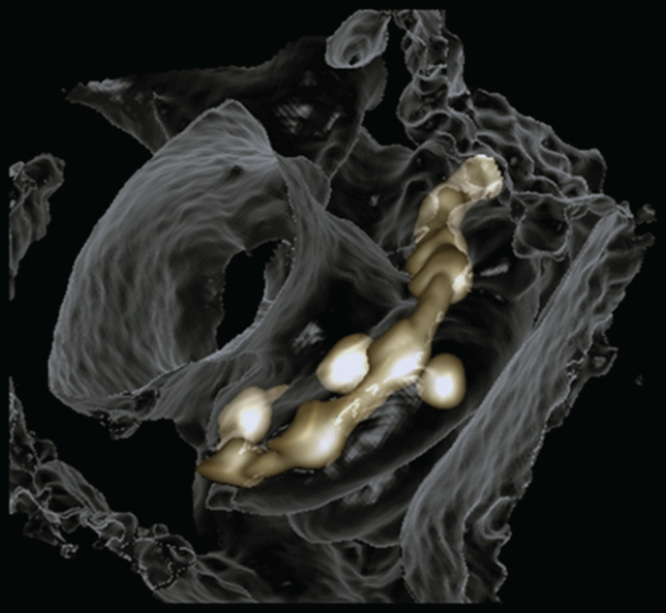
**3D reconstruction of cone-beam computed tomography (CBCT) images showing spatial correlation between the electrode contacts and the osseous cochlear modiolus.**

By evaluating the A values (Figure 4), height of the cochlea, and the insertion depth for the first 360° insertion angle there was a linear correlation between the A value and cochlea height, and the A value and insertion depth for the first 360° insertion angle (Figure 5). However, there was no correlation between the full insertion depth of the electrode and insertion angle for the full insertion depth of the electrode (Table III). The spatial correlation between the electrode contacts and osseous cochlear modiolus was depicted by 3D reconstruction (Figure 6).

**Table III.** **Insertion angle and insertion depth of the electrode arrays with number of contact pairs outside the cochlea.**

Sample no./electrode type	Insertion depth for first 360° insertion angle (mm)	Full insertion depth of electrode (mm)	Insertion angle for full insertion depth of electrode (°)	No. of contact pairs extracochlear
1/Standard	18.8	20.4	414	3
2/36 channel	18.6	18.6	340	13*
3/FLEX28	19.8	21	520	1
4/FLEX28	19.9	21	429	1
5/Standard	21.2	20.4	367	3
6/Standard	18.2	20.4	468	2

*One line of contacts.

## Discussion

In comparison with reports in the literature, the present experimental set-up of CBCT demonstrated the cochlear structures and CI electrode arrays with the highest spatial resolution, less metallic artefacts, and excellent contrast, and is capable of showing the critical landmarks of the cochlea clearly [1, 2, 4, 5]. This is valuable in clinical practice in both the preoperative evaluation of the fine intracochlear structures and intraoperative or postoperative evaluation of the position of the electrode array. Preoperative detailed evaluation of the intracochlear structures will help the surgeon to plan the surgery well and to avoid any intraoperative surprises, for example, fibrous tissue or intracochlear bone formation. Intra- and postoperative evaluations will help the surgeon to see if there is any electrode migration between the scalae, which is a direct measure of potential intracochlear trauma and has a negative impact on hearing preservation. The other reason for intra- or postoperative evaluations is to check if there is any electrode kink, buckle or tip folding and take a countermeasure by switching off the contacts in that region to improve the patient’s performance. In an extreme case, it might be necessary to replace the electrode array exhibiting fatal failure with a new one in timely fashion to avoid revision surgery later.

Guldner et al. reported that a high rate of artefacts (50%) made it extremely difficult to predict the inserted scale, especially when evaluating the intracochlear position in the medial and apical turn of the cochlea using a 3D Accuitomo device (J. Morita, Kyoto, Japan) with a Toshiba D-051 tube (Toshiba, Otawara, Japan) to generate X-rays [12]. Our understanding in that study is that the resolution was limited by the pixel size, imaging geometry, and number of images, which was unable to distinguish the fine structures in the cochlea, especially in the higher coils [13]. A suitable adjustment on the γ curve of the original images that were displayed using the Romexis Viewer Demo program is another attributable factor that suppressed metal artefacts on the images in the present study.

The observed correlation between the A value and cochlear height suggested that human cochleae have a constant ratio between the basal turn diameter and the height. The existence of linear correlation between the A value and the actual insertion depth for the first 360° insertion angle in the present study is in accordance with previous reports [6, 7, 8, 14]. This linear correlation also supports the method of using A value to select the electrode model that was established by MED-EL [15]. The finding that there is no correlation between the full insertion depth of the electrode and insertion angle for the full insertion depth of the electrode indicates that the same full insertion depth of the electrode can end at any angle (see Table III, columns 3 and 4). This supports the proposition that variation in cochlear size (especially the cochlear length) to accommodate the electrode does exist. The new experimental electrode with a 36-contact array did not follow the ‘rule,’ as the electrode was closer to the modiolus wall in the basal turn and was somehow not deeply inserted as a result of increased rigidity caused by shorter contact separation in comparison with the other two electrode arrays. The electrode to modiolus wall distance measurement shown in the present study demonstrated that it is possible to carry out such measurements in clinical practice. The landmark used to measure the distance from the center of the contact to the modiolus wall is different from the method reported by Esquia Medina et al., who measured the distance between the center of the contact and the center of the modiolus axis [8].

For the technique to become feasible clinically, the issue of motion blur needs to be addressed. Motion blur originates from two sources, movement of the patient during scanning and movement of the radiation source during the finite length exposure pulse. The latter can be mitigated by shortening the exposure pulse, at the expense of increased noise, or by slowing down the rotation speed of the imaging apparatus. However, this will increase the likelihood of patient motion. This is not a problem when the system is used in the operating theater. In the case of working in the outpatient clinic, patient motion could potentially be reduced by immobilizing the patient using custom-made support mechanisms such as vacuum cushions. However, developing all these steps will not take a long time.

In conclusion, the present experimental set-up of high spatial resolution CBCT showed advantages of demonstrating the critical landmarks of the cochlea in identifying the position of intracochlear electrode contacts. Also the cochlear measurements demonstrated in this study are in good agreement with previous studies using micro-CT, and corrosion cast shows the efficacy of the presented imaging set-up in clinical practice. For future designing of advanced CBCT, increase of frame number to 900 and avoiding rotation of the C-arm during scanning can produce images with higher quality than with current protocols. Although adding up image frames will increase the radiation (maximum value = 138 µSv), the effective dose might be reduced by keeping the voltage and current at an optimized low level.
